# Sono-piezoelectric cues regulate neuroinflammatory reflex-arc-mediated α7nAChR-P2RX7 axis to dampen osteoarthritis-correlated pain with osteoarthritis attenuation

**DOI:** 10.7150/thno.125686

**Published:** 2026-01-30

**Authors:** Qiuling Zhong, Junqi Xie, Maocheng Zuo, Chuanan Liao, Qiuxia Peng, Simeng Yu, Pan Hu, Yuanyuan Liu, Li Zheng, Kun Zhang, Zhenhui Lu, Jinmin Zhao

**Affiliations:** 1 Guangxi Engineering Center in Biomedical Materials for Tissue and Organ Regeneration, Collaborative Innovation Centre of Regenerative Medicine and Medical BioResource Development and Application, Guangxi Key Laboratory of Regenerative Medicine, The First Affiliated Hospital of Guangxi Medical University. No. 6 Shuangyong Road, Nanning, Guangxi 530021, China.; 2 Life Sciences Institute, Guangxi Medical University. No. 22 Shuangyong Road, Nanning, Guangxi 530021, China.; 3 Department of Orthopaedics and Central Laboratory, Sichuan Academy of Medical Sciences, Sichuan Provincial People's Hospital, School of Medicine, University of Electronic Science and Technology of China, No. 32, West Second Section, First Ring Road, Chengdu, 610072, Sichuan, China.; 4 Ultrasound in Cardiac Electrophysiology and Biomechanics Key Laboratory of Sichuan Province, Sichuan Academy of Medical Sciences, Sichuan Provincial People's Hospital, School of Medicine, University of Electronic Science and Technology of China, No. 32, West Second Section, First Ring Road, Chengdu, 610072, Sichuan, China.; 5 Guangxi Key Laboratory of Bioactive Molecules Research and Evaluation, Pharmaceutical College, Guangxi Medical University. No. 22 Shuangyong Road, Nanning, Guangxi 530021, P. R. China.; 6 Department of Orthopaedics Trauma and Hand Surgery, The First Affiliated Hospital of Guangxi Medical University. No. 6 Shuangyong Road, Nanning, Guangxi 530021, China.

**Keywords:** Osteoarthritis, Osteoarthritis-correlated pain, Sono-piezoelectric ZnO nanoparticles, α7nAChR-P2RX7 axis, Neuroinflammatory Reflex-arc

## Abstract

**Background:** Osteoarthritis (OA) characterized by progressive cartilage degeneration and chronic pain is hindered by the vicious inflammation-pain cycle. Nonsteroidal anti-inflammatory drugs (NSAIDs) can only alleviate clinical symptoms. Although electrical stimulation of the vagus nerve has achieved some results, the toxicity of the electrodes and the secondary damage caused by dismantling limited its clinical application.

**Methods:** A “sono-piezoelectric-bioelectricity-neuroimmune” cascade modulation strategy based on ultrasound-driven piezoelectric ZnO nanoparticles was established to attenuate osteoarthritic neurogenic inflammation and pain with inhibited cartilage degradation.

**Results:** The “sensory neuron-cholinergic anti-inflammatory pathway” reflex arc was activated by the dynamically and spatially-temporally programmed sono-piezoelectric field (0.7 V/36 μA peak) within deep joint tissues by targeting α7nAChR-P2RX7 neuroimmune axis. Consequently, our sono-piezoelectric neuroimmune modulation strategy significantly up-regulated α7nAChR expression, and synchronously inhibited pain mediator CX3CL1, opposed macrophage infiltration, inhibited P2RX7-mediated IL-1β/IL-6 inflammatory storm, restored IL-1β-injured chondrocyte activity and migration capacity, activated stromal genes (Col2a1) for matrix synthesis, and inhibited cartilage degradation-related MMP13 expression. All these actions re-established the balance of glycosaminoglycan (GAG)/deoxyribonucleic acid (DNA) metabolism to remodel joint immune homeostasis, and activated the cholinergic pathway to break the vicious cycle of “inflammation-pain”, which restored mechanical pain threshold (Von Frey) and weight-bearing capacity to near-normal levels and reconstructed tidal structures in a rat OA model.

**Conclusions:** Our pioneering “sono-piezoelectrical signal-neural reflex-immunomodulation” cascade strategy for regulating neuroinflammatory reflex-arc-mediated α7nAChR-P2RX7 axis provide deep insights into OA-represented neuroinflammatory diseases.

## Introduction

Osteoarthritis (OA) is a highly disabling degenerative disease. Its core pathology involves progressive degradation of articular cartilage and activation of synovial inflammation, accompanied by abnormal sensitization of pain signaling pathways 1, 2. These pathological changes lead to extreme pain and severely limit daily functioning, and the vast OA population (over 654 million incidence rate per year) poses a major public health burden. Current non-surgical intervention relies on analgesics and non-steroidal anti-inflammatory drugs (NSAIDs). Although these provide temporary pain symptom relief, they fail to reverse cartilage degradation. Long-term use also brings risks of gastrointestinal complications, cardiovascular events, and dependency 3. Surgical approaches such as autologous chondrocyte implantation (ACI) and osteochondral grafting are effective for focal cartilage defects but show limited efficacy in cartilage damages 4, 5. Additionally, allografts face inherent limitations, including donor scarcity, immune rejection, and disease transmission risks 6. All these unsatisfactory outcomes can be attributed to the complex neuroimmune microenvironment in OA, because the polarization imbalance of synovial macrophages and the upregulation of pain-related ion channels (e.g., TRPV1, Nav1.8) in the dorsal root ganglion (DRG) have been identified as critical nodes driving the inflammation-pain vicious circle in OA pathology 7. It determines that neuron-inflammation crosstalk in OA can serve as a target for attenuating pain and OA with satisfactory cartilage repair.

Bioelectrical signals play a critical role in the regulations of nerve and immune homeostasis through the cholinergic anti-inflammatory pathway (CAP) 8, 9. Mechanistically, vagus nerve-derived acetylcholine (ACh) binding to the α-7 nicotinic acetylcholine receptor (α7nAChR) on innate immune cells constitutes an “inflammatory reflex” circuit 10, 11, which effectively inhibits pro-inflammatory cytokines (including TNF-α, IL-1β, and IL-6) through inhibiting NF-κB nuclear translocation and inflammasome activation 12. Meanwhile, α7nAChR has been shown to be a pivotal mediator of OA injurious information processing 13. Dysregulated expressions of this receptor in synovial macrophages and dorsal root ganglion neurons drives TRPV1 channel sensitization with enhanced Nav1.8 sodium currents via the Jak2-STAT3 pathway to exacerbate mechanically abnormal pain and thermal nociceptive sensitization 14, which provides a theoretical opportunity for synergistic therapeutic strategies for simultaneous intervention of inflammation and pain 15. Unfortunately, the efficient and precise delivery of therapeutic electrical current doses into deep joint target tissues for activating bioelectrical signals remains challenging, e.g., electrodes-arised trauma, infection, foreign body rejection and contact impedance instability, high impedance tissues-arised current attenuation and non-focused current-arised non-targeted tissue activation 16-18, akin to other minimally-invasive means 19.

Piezoelectric dynamical process based on piezoelectric materials are expected to provide precise and stable bioelectrical signals or stimuli 20-23. Especially, sono-piezoelectric signals activated by ultrasound (US) pressure feature exceptional spatial-temporal programmability 24-27, which have been used to promote the differentiation and proliferation of stem cells for nerve trauma repair and bone generation 28-32. Enlightened by them, here we proposed a “US-driven piezoelectricity-neuromodulation” to break through the critical bottleneck of the vicious cycle of inflammation and pain and create a dynamically programmable electrical microenvironment within joint tissues for OA-arised pain alleviation and OA recession with inhibited cartilage degradation. Differing from light sitmuli 33, 34, the millimeter spatial focusing and millisecond timing programming capabilities of US 35, enable deep tissue penetration and targeted piezoelectric activation without damages to surrounding neurovascular structures, and coincidently unlock rhythmic electrical signals that mimic the dynamics of physiological mechanical loads, thereby potentially preventing cellular electrical adaptation. Crucially, the linear dependence of piezoelectric charge output on US intensity enables non-invasion, real-time, and on-demand delivery of therapeutic electrical signals. Among the numerous piezoelectric materials, zinc oxide nanoparticles (NPs) have been attracted much attention due to the exceptional piezoelectric properties owing to their non-centrosymmetric crystal structures 36-38.

In this study, morphologically-homogeneous cubic-phase ZnO piezo-electric NPs were obtained via microfluidics and characterized with excellent piezoelectricity, biocompatibility and tunable semiconductor properties 39, 40, enabling non-invasive, *in*-*situ*, on-demand, dynamic piezoelectric fields (peak output of 0.7 V/36 μA) in deep articular tissues that favored damaged cartilage regeneration. This strategy demonstrated that localized sono-piezoelectric fields could target and activate α7nAChR in sensory neurons and synovial tissues in the joint cavity, specifically initiating the CAP. The centerpiece of this reflex arc lay in the bidirectional regulation through the α7nAChR-P2RX7 axis. On the one hand, pain mediator CX3CL1 expression and macrophage infiltration were inhibited in DRG to block nociceptive signaling. On the other hand, pro-inflammatory factors (IL-1β, IL-6) and matrix degrading enzyme (MMP13) expression were down-regulated in synovial tissue to reverse immune imbalance. On the anterior cruciate ligament tenotomy (ACLT)-constructed rat OA model, a single intra-articular injection combined with US intervention could achieve 64% down-regulation of synovial IL-1β expression and 82% improvement in pain behavioral scores. Collectively, this strategy provided a novel non-invasive, programmable and precise physical intervention strategy for OA that integrated highly effective anti-inflammatory, long-lasting analgesic potentials with reversed cartilage degradation, overcoming the invasiveness inherent in implantable electrical stimulation.

## Results and Discussion

### Preparation, characterizations and sono-piezoelectric property evaluations of ZnO NPs

**Figure 1:**
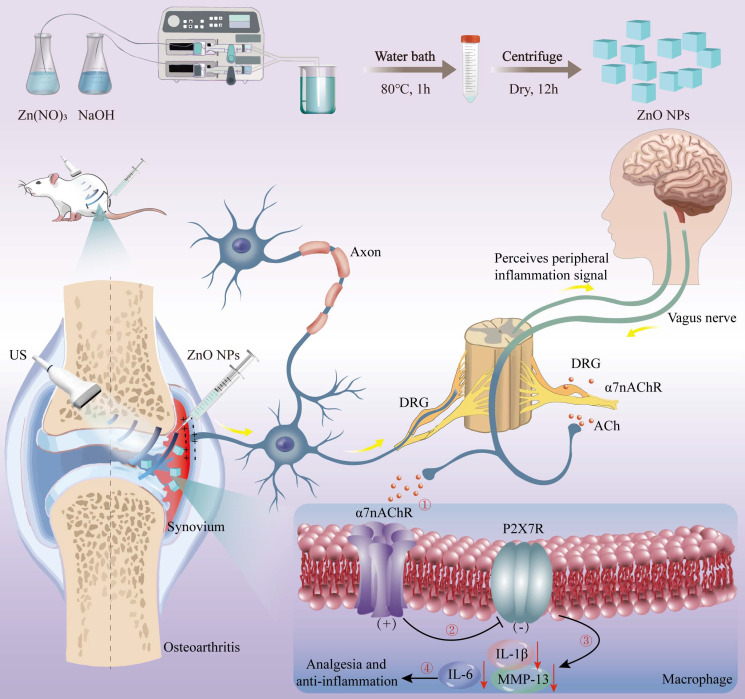
Schema of piezoelectric ZnO NPs synthesis and OA treatment via neuroinflammatory reflex-arc mediated α7nAChR-P2RX7 axis.

ZnO NPs were obtained through the continuous flow synthesis method using a microfluidic spiral reactor 41, where a Y-shaped microfluidic reactor was used (**Figure 1**). Briefly, Zn(NO_3_)_2_ solution reacted with NaOH solution to generate Zn(OH)_2_ intermediates and then selectively adsorbed two OH^-^ into [Zn(OH)_4_]^2-^, followed by alkaline environment-driven hydrolysis of [Zn(OH)_4_]^2-^ into ZnO NPs. Herein, the selective adsorption of OH^-^ on the cubic crystal phase surface inhibits axial growth, which combined with high shear rate to constrain the nuclei self-assembly and generate isotropic concentric cubic particles with significantly-enhanced piezoelectric polarization response 42. The spatiotemporal precision of microfluidics decoupled the nucleation and growth kinetics, dramatically shortening the synthesis cycle compared to hydrothermal methods, which provides a standardized piezoelectric nanoplatform for neuroinflammatory reflex arc studies 43.

**Figure 2:**
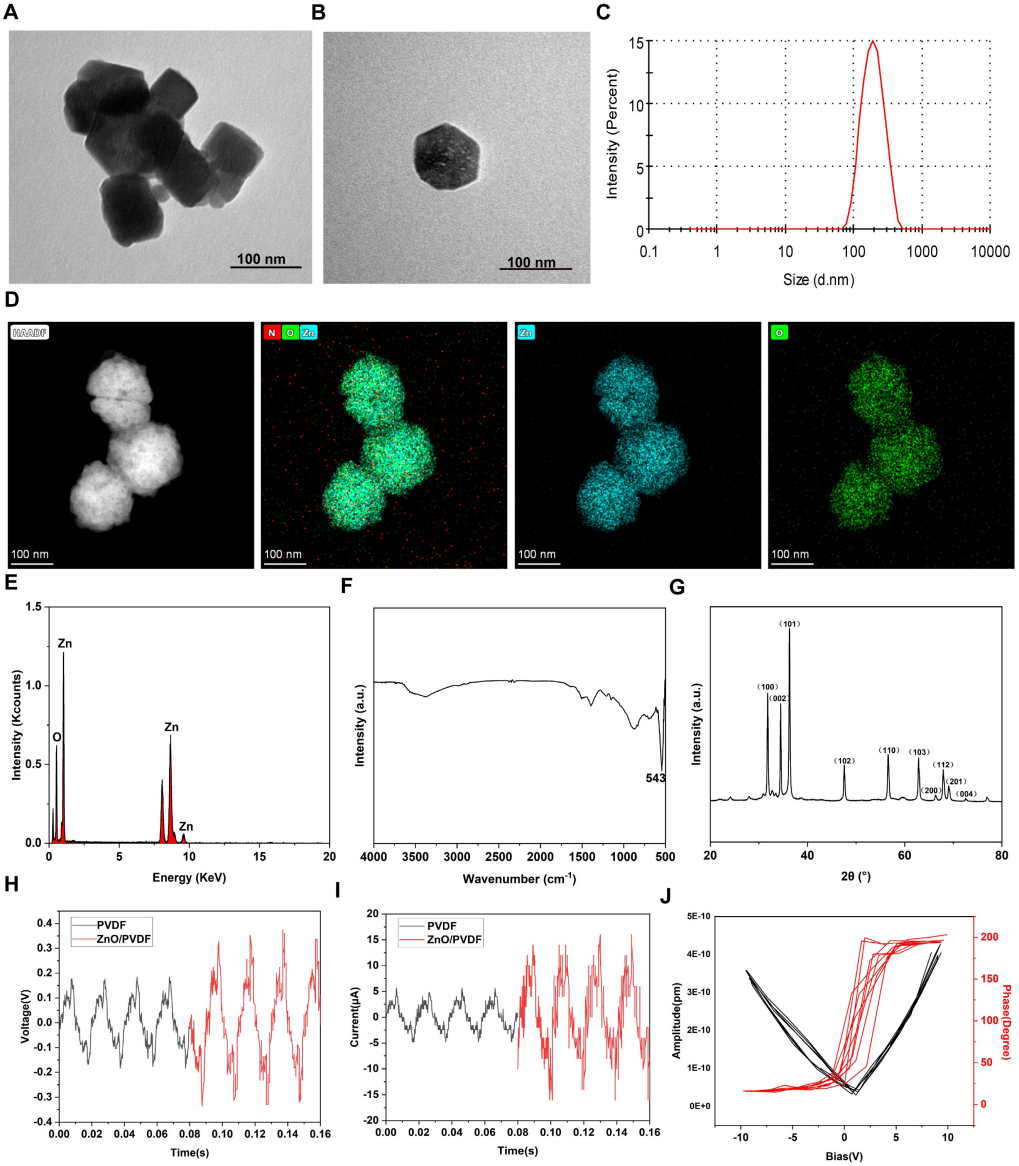
** Synthesis, characterization and piezo-electric response inspection of ZnO NPs.** (A-B) Transmission electron microscopy (TEM) images of ZnO NPs (Scale bar: 100 nm). (C) Dynamic light scattering (DLS)-determined particle size of ZnO NPs. (D) High-angle annular dark-field scanning transmission electron microscope (HAADF-STEM) for elemental mapping of Zn and O in ZnO NPs (Scale bar: 100 nm). (E-G) Energy dispersive spectrum (EDS) (E), fourier transform infrared spectroscopy (FTIR) spectrum (F) and X-ray diffraction (XRD) pattern (G) of ZnO NPs. (H,I) Voltage (H) and current (I) generated by ZnO/PVDF piezoelectric thin films. (J) Piezoresponse force microscopy (PFM)-determined spectra of ZnO NPs.

The morphology, chemical composition, crystal structure, and piezoelectric properties of synthesized ZnO NPs were inspected using multiscale techniques (**Figure 2**). ZnO NPs exhibit a uniformly-dispersed cubic structure with an average particle size of around 100 nm (**Figure 2A-B**), suggesting that the microfluidic technology can achieve precise morphology modulation. The hydrodynamic diameter of ZnO NPs in the aqueous phase is determined to be around 200 nm, which satisfies the bio-delivery requirements and consistent with biomedical applications (**Figure 2C**). The spatial distributions of Zn (blue) and O (green) signals in ZnO NPs completely overlap, confirming the homogeneity of the spatial chemical distribution (**Figure 2D**). In addition, ZnO NPs only contain Zn and O atoms, demonstrating the completeness of the reaction and high chemical purity (**Figure 2E**). ZnO NPs show strong absorption bands in the region from 400 to 800 cm^-1^, where the characteristic peak at 543 cm^-1^ was attributed to the Zn-O tetrahedral telescopic vibration, confirming the formation of ZnO (**Figure 2F**). The as-prepared ZnO NPs show sharp diffraction peaks, which are strictly aligned with the positions of the characteristic peaks of the standard diffraction spectrum of the fibrillar zincite structure ZnO, indicating the high crystallinity (**Figure 2G**).

Subsequently, we confirmed the piezoelectric properties of ZnO NPs through electrical output characteristic inspections and microscopic piezoelectric response detection. Specifically, the voltage and current outputs generated by the ZnO/PVDF composite film under mechanical impact were recorded (**Figure 2H,I**). The voltage peak reaches 0.7 V, representing a 300% enhancement over the pure PVDF film, and concurrently a considerably-elevated peak current at 36 μA is obtained. This significant enhancements in voltage and current effectively facilitated the interfacial charge separation process. Further microscale observations directly reveal the piezo-responsive behavior of the nanoparticles (**Figure 2J**). The amplitude-voltage curves exhibit a typical butterfly-shaped hysteresis return line characteristic with an amplitude difference ΔA of 1.2 nm. Meanwhile, the phase angle undergoes a 180-degree flip near the zero-volt bias. The amplitude profile together with the phase flip phenomenon confirms the reversible switching mechanism of the iron-electrode polarization domains in the material. Such excellent piezoelectric properties provide a solid mechanistic basis for understanding the physical nature of US-driven electrical signal output, which are anticipated to offer efficient remote charge carriers for US-driven neuromodulation such as α7nAChR pathway activation.

### US-driven sono-piezoelectric ZnO NPs enhanced the viability of IL-1β-induced chondrocytes *in vitro*

**Figure 3:**
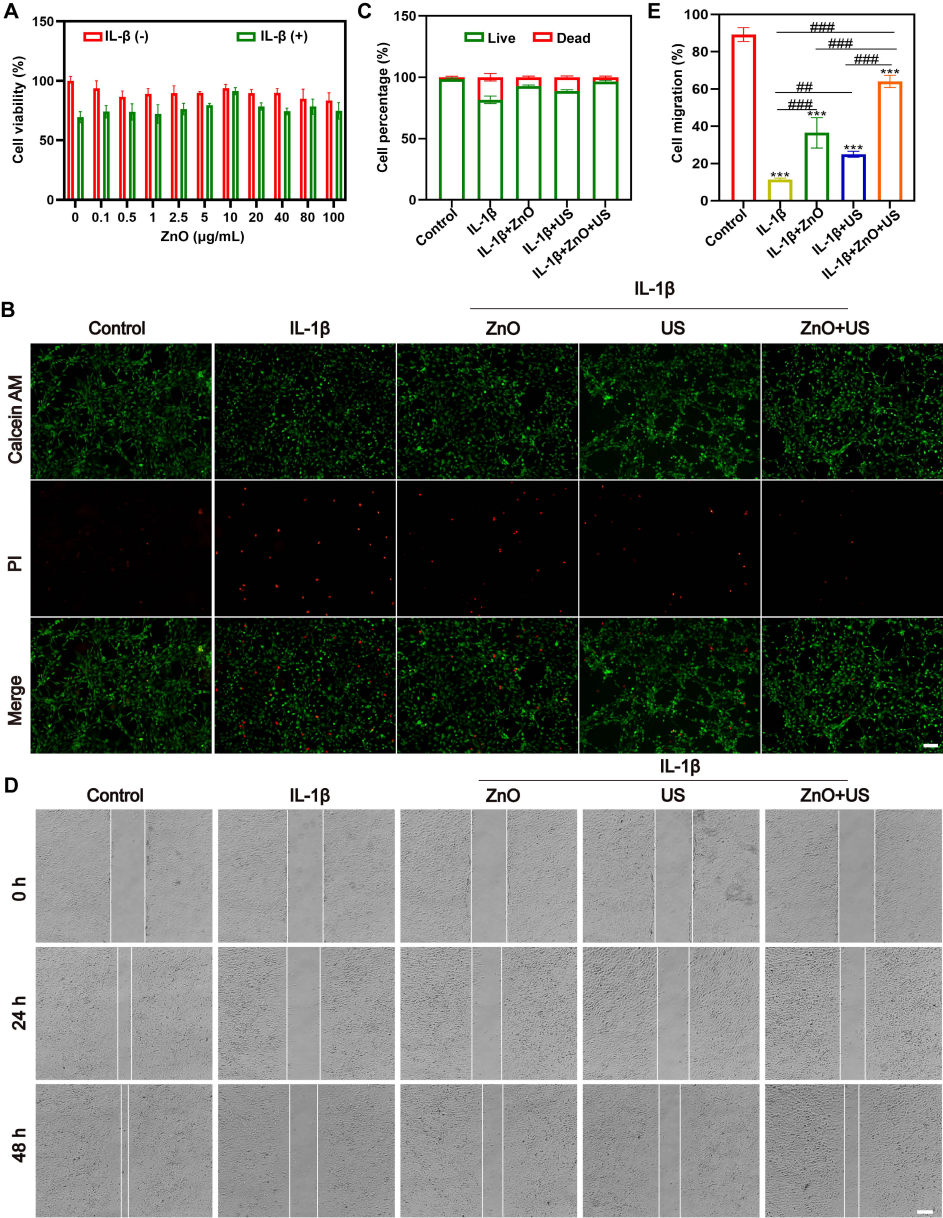
** Biocompatibility of ZnO NPs in the presence of US stimulation.** (A) Relative viabilities of chondrocyte incubated with ZnO NPs at different concentrations in the presence or absence of IL-1β. (B) Fluorescence images of chondrocytes stained with Calcein AM/PI for discerning live/dead cells after different treatments (Scale bar: 200 μm); and (C) Semi-quantitative analysis of chondrocyte viability. (D) Scratch images of chondrocytes after different treatments (Scale bar: 200 μm) and (E) Semi-quantitative analysis of migration rate. Data is mean ± SD (n = 3). ***p < 0.001 *vs.* Control; ^##^*p <* 0.01, ^###^*p <* 0.001 for intergroup comparisons.

The chondrocytes used was polygonal or near circular in shape, which is the characteristic morphology of hyaline chondrocytes (**Figures S1**). To figure out whether sono-piezoelectric ZnO NPs activated by US could effectively mitigate the damages to chondrocytes from the inflammatory microenvironment and promote their functional recovery 44, we systematically evaluated the biocompatibility and repair potential on interleukin-1β (IL-1β)-induced inflammatory chondrocytes. Biosafety assay firstly established a safety threshold for ZnO NPs (**Figure 3A**), wherein 10 μg/mL ZnO NPs was identified as a safe therapeutic window for subsequent experiments since cell viability maintains >90% at this concentration even under inflammatory conditions (+IL-1β). The optimization of ultrasound parameters for piezoelectric ZnO NPs treatment was also screened. It demonstrated that US intensity at 0.35 W/cm^2^ for 90 s resulted in the highest activity (**Figure S2**). Next, calcein AM/PI live-dead cell double staining experiments revealed that IL-1β stimulation led to PI-positive dead cells, and single ZnO NPs or sonication intervention only partially reversed this apoptotic trend (**Figure 3B,C**). In contrast, ZnO NPs in the presence of US (0.35 W/cm^2^, 90 s) demonstrated a synergistic protective effect, restoring the density of live cells to near-normal levels, reliably confirming the significant inhibitory effect of piezoelectric activation on inflammation-induced cell death 45.

Subsequently, we further explored its functional repair ability, and scratch migration assay showed that IL-1β severely impaired chondrocyte migration, with the healing rate decreased to ~10% (**Figure 3D,E**), and single treatment group (ZnO NPs or US) failed to prominently improve it. In a sharp contrast, US-driven ZnO NPs treatment group demonstrated a strong pro-migration effect, with a substantial increase in wound healing rate to nearly 80% of the normal group. With integrating the multidimensional evidences of cell activity, viability and migration function, the piezoelectric effect of US-activated ZnO NPs significantly alleviated IL-1β-mediated inflammatory damages and enhanced the repair potential of chondrocytes by coordinating the anti-apoptotic pathway with cytoskeletal dynamics. This strategy provided a novel sono-piezoelectric-catalyzed intervention route to target and regulate the cartilage matrix degradation-regeneration imbalance in OA.

### US-driven sono-piezoelectric ZnO NPs decreased inflammation and maintained the phenotype of IL-1β-induced chondrocytes *in vitro*

**Figure 4:**
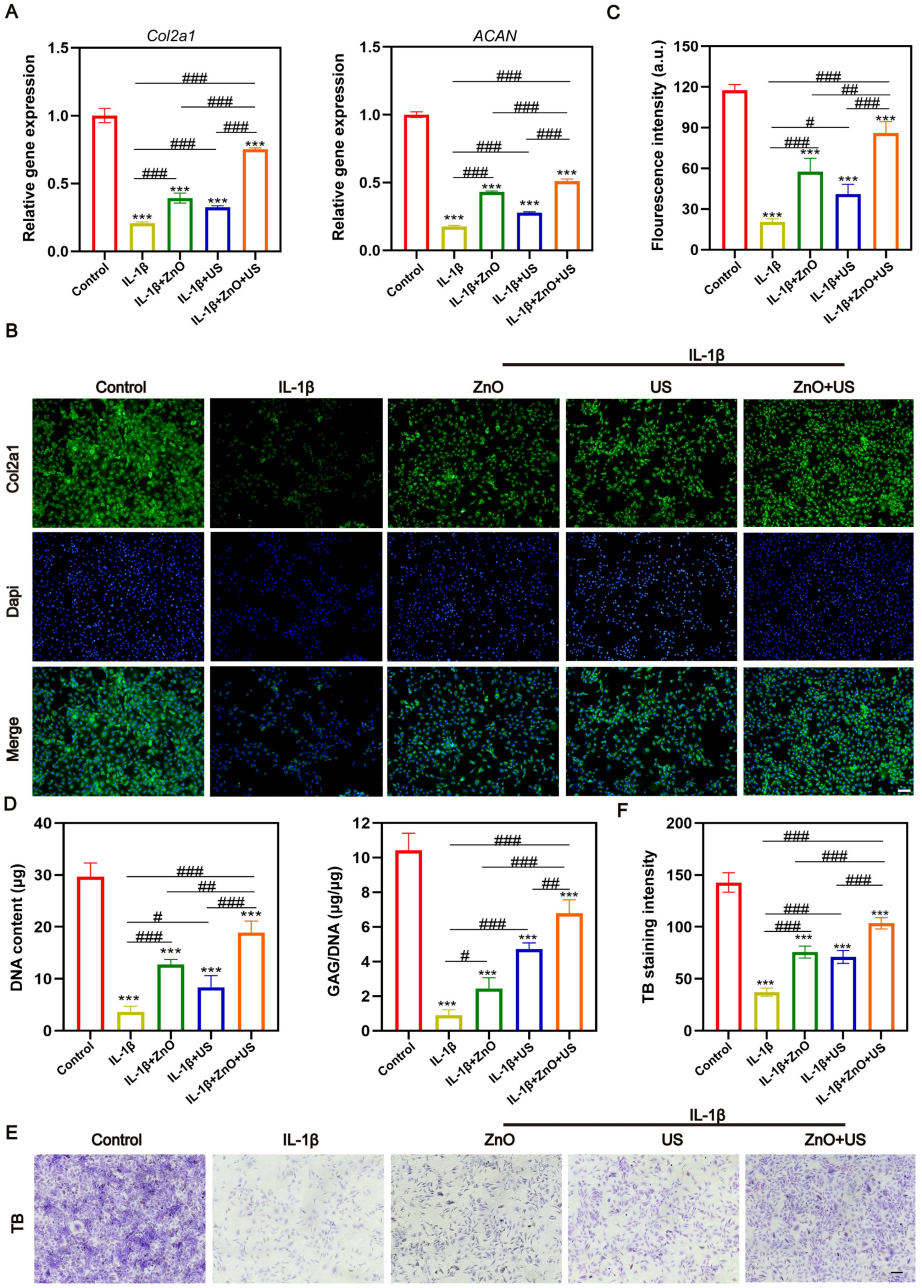
** Anti-inflammatory and chondrocyte phenotype maintainced effects of ZnO NPs under US stimulation.** (A) mRNA expression levels of *Col2a1* and *ACAN*. (B,C) Immunofluorescence images (B) and semi-quantitative florescence intensity (C) of chondrocytes stained with COL2a1 after different treatments (Scale bar: 200 μm). (D) Statistical analysis of DNA content and GAG to DNA ratio in chondrocytes after different treatments. (E,F) Immunohistochemical staining images (E) and semi-quantitative TB intensity (F) of chondrocytes stained with methylamine blue after different treatments (Scale bar: 200 μm). Data is mean ± SD (n = 3). ***p < 0.001 *vs.* Control; ^#^*p <* 0.05, ^##^*p <* 0.01, ^###^*p <* 0.001 for intergroup comparisons.

To resolve the action mechanism of sono-piezoelectric ZnO NPs on chondrocyte phenotype maintenance 46, we assessed the extracellular matrix (ECM) synthesis and inflammation modulation efficacy. After chondrocytes were subjected to IL-1β inflammatory stimulation,* Col2a1* and *ACAN* gene expression plummeted to 21% and 18% of the normal group, respectively (**Figure 4A**), confirming that the inflammatory environment severely compromised cartilage matrix synthesis. Remarkably, the US-activated ZnO NPs-treated group restored Col2a1 expression to 80% of the normal level and elevated ACAN expression by 50%, which were much higher than those in single ZnO NPs or US group. Furthermore, immunofluorescence staining revealed that the dispersion of COL2a1 protein signals was attenuated in the IL-1β group, whereas the ZnO+US group showed a dense periplasmic fiber network structure (**Figure 4B**) with the highest fluorescence intensity (**Figure 4C**), suggesting that the piezoelectric stimulation was effective in reversing the impediment of collagen II synthesis 47. Additionally, IL-1β stimulation expedited more secretions of pro-inflammatory cytokines IL-6 and TNF-α, while the ZnO+US treatment significantly inhibited IL-6 and TNF-α secretion at both gene (**Figure S3A and D**) and protein (**Figure S3B, C, E and F**) levels and successfully alleviated inflammation.

We further examined the GAG/DNA ratio, a pivotal metric to quantify matrix metabolic homeostasis (**Figure 4D**). IL-1β-induced chondrocytes exhibited severe GAG loss, with a 91% reduction in the ratio, however, the ZnO+US group elevated GAG retention to 65% of the normal group, which was significantly higher than that of the control group. This result was highly consistent with the toluidine blue staining features (**Figure 4E,F**), and only a weak blue-purple signal was seen in the inflammation group, suggesting proteoglycan depletion. On the contrary, the ZnO+US group presented deeply stained heterogeneous regions with a quantitative increase of 1.8-fold in staining intensity, visually confirming that piezoelectric stimulation restored chondrocyte anabolic function by re-establishing ECM synthesis-degradation homeostasis. To understand the mechanism, MMP13 as a OA marker capable of degrading collagen and proteoglycan was monitored, and the ZnO+US group significantly inhibit the transcription and translation of MMP13, resulting in the lowest MMP13 level (**Figure S4**). Collectively, these results suggest that US-driven ZnO piezoelectric nanoparticles regained phenotypic stability of damaged chondrocytes through dual pathways, i.e., inflammatory factor storm inhibition and activation of matrix synthesis genes. Thus, the dual repair efficacies armed on US-driven piezoelectric ZnO NPs successfully achieved the central goal of chondrocyte phenotypic remodeling at the cellular level by reversing IL-1β-induced anabolic inhibition and blocking the catabolic cascade. This finding not only reveals the reprogramming ability of piezoelectric stimulation on ECM homeostasis but also provides a cellular-molecular basis for the subsequent systemic anti-inflammatory mechanism mediated by the neural reflex arc^48^, ^49^.

### US-driven sono-piezoelectric ZnO NPs attenuated OA progress in OA rats

**Figure 5:**
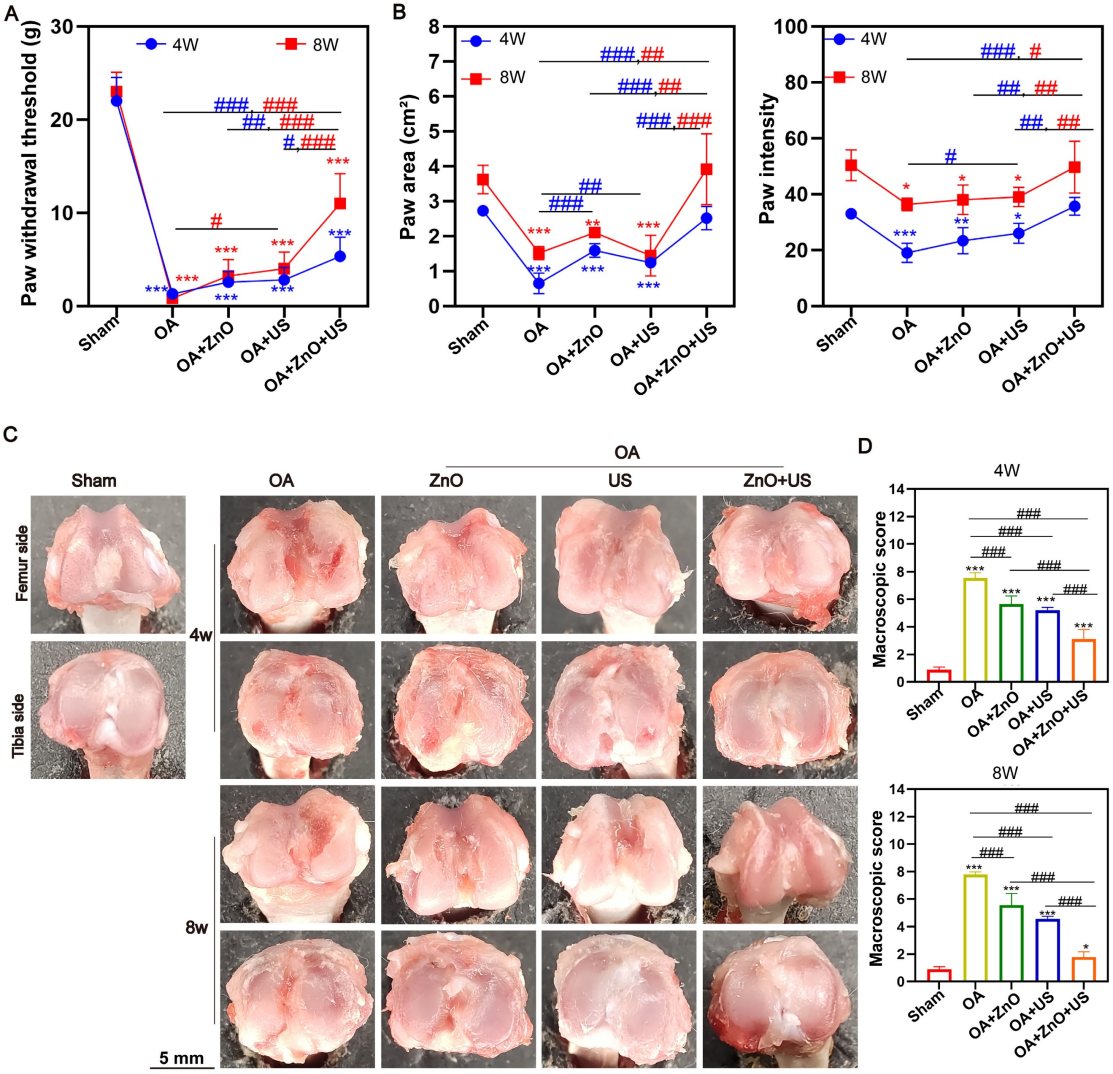
***In vivo* OA progression suppression by US-driven piezoelectric ZnO NPs.** (A,B) Von Frey test (A) and animal gait analysis (B) after different treatments for 4 and 8 Weeks. (C,D) Gross observation (C) and macroscopic scores (D) of knee joint after different treatments for 4 and 8 Weeks. Data is mean ± SD, (n = 5). *p < 0.05, **p < 0.01, ***p < 0.001 *vs.* control; ^#^*p <* 0.05, ^##^*p <* 0.01, ^###^*p <* 0.001 for intergroup comparisons.

After confirming the significant effect of US-driven piezoelectric ZnO NPs on interleukin-1β-induced chondrocyte inflammation and phenotypic maintenance, we further performed US-activated ZnO NPs in a ACLT-constructed rat OA model with critical-sized osteochondral damages to evaluate their therapeutic effects *in vivo*. The model was, which effectively simulated the pathological process and pain response of OA. To comprehensively measure the therapeutic potential, we integrated multimodal behavioral analysis and joint morphology assessment (**Figure 5**). Firstly, we quantitatively assessed mechanical nociceptive sensitization in rats using the Von Frey experiment, which is a central feature of OA pain. Results showed that the mechanical retraction threshold of foot drastically declined in the OA model group compared to the sham-operated group, indicating a significantly higher nociceptive sensitivity (**Figure 5A**). Notably, the pain tolerance of rats treated with ZnO NPs in combination with US (ZnO+US group) showed a tendency to be superior to that of ZnO NPs alone or US alone at 4 weeks, and was further significantly enhanced at 8 weeks, with thresholds close to normal levels, strongly suggesting OA-related pain relief.

Immediately thereafter, we performed non-invasive quantitative analysis of gait parameters under spontaneous walking status utilizing an animal gait analysis system to assess joint function recovery (**Figure 5B**). Here key indicators reflecting weight-bearing capacity and gait stability: mean footprint area and mean strength (correlated with plantar pressure) were underlined. Rats in the OA group showed significantly reduced footprint area and strength, suggesting a reduction in weight-bearing of the affected limb and shorter touchdown time due to pain and joint dysfunction. In contrast, rats in the ZnO+US group showed a significant enhancement of both parameters after 8 weeks, and their footprint characteristics were closer to those of normal rats, whereas the application of ZnO NPs or US alone only received partial improvement. This result, together with Von Frey test, confirms the synergistic benefits of ZnO+US therapy in improving motor function and reducing pain in OA rats 50.

Gross morphological observations of rat knee joints after 4 and 8 weeks post-treatment were proceeded to visually assess the degeneration degree of articular cartilage (**Figure 5C**). The articular surface in the normal knee surgery group was smooth, intact and shiny, while the articular surface in the OA group lost luster, and became rough and uneven with evident vascular opacities, and the degeneration progressively worsened over time. Although the use of ZnO NPs or US alone could reduce the degree of degeneration to some extent, the articular surfaces were still rough. Fortunately, the articular cartilage surface gradually regained its luster in the ZnO+US group, with the degeneration area being significantly reduced, and the joint structure appearance being closer to the healthy state. Quantitative analysis showed that the ZnO+US group obtained the lowest degeneration score at the endpoint of treatment (8 weeks) (**Figure 5D**). Appealingly, US-driven piezoelectric ZnO NPs in the joint cavity can effectively slow down or even reverse the morphological progression of OA.

**Figure 6:**
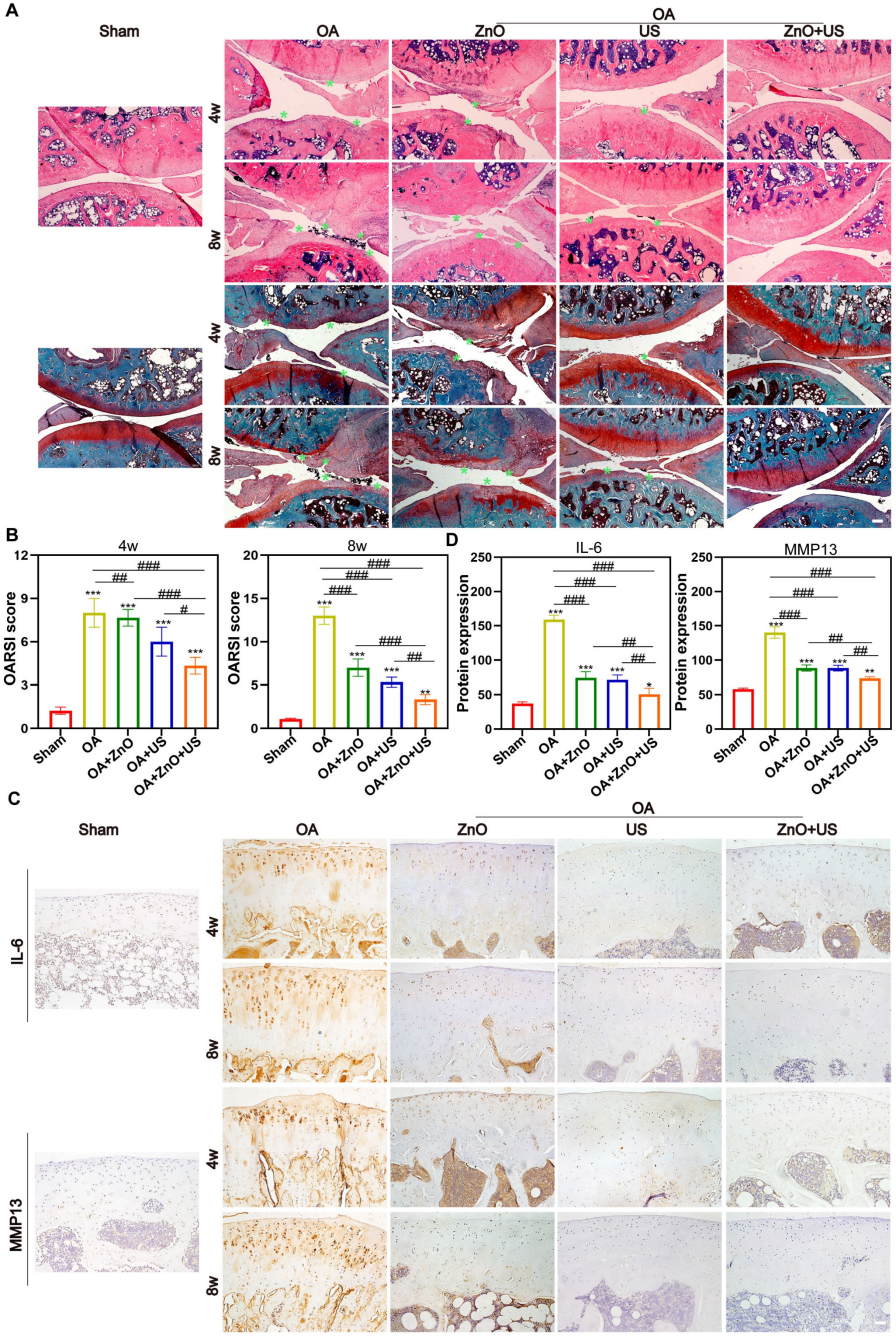
***In vivo* maintenance of cartilage structure and ECM by US-driven piezoelectric ZnO NPs.** (A) H&E and Safranin O-Fast green staining (Scale bar: 200 μm). (B) OARSI scores of knee joints at week 4 and 8 (green arrow indicates cartilage erosion). (C) Immunohistochemical staining images for detecting IL-6 and MMP13 at 4 and 8 weeks (Scale bar: 50 μm). (D) Semi-quantitative analysis of protein expression in (C). Data is mean ± SD, (n = 5). *p < 0.05, **p < 0.01, ***p < 0.001 *vs.* control; ^#^*p <* 0.05, ^##^*p <* 0.01, ^###^*p <* 0.001 for intergroup comparisons.

Subsequently, we further systematically evaluated the *in vivo* therapeutic mechanisms of sono-piezoelectric ZnO NPs under US activation to comprehensively validate their reparative effects on joint degeneration. Double staining with H&E and Safranin O-Solid Green showed that the cartilage surface in the OA group was severely damaged, with uneven matrix staining and progressive structural collapse, and the damage continued to deteriorate over time (4 weeks to 8 weeks) compared to that in the normal group with structurally intact and clearly delineated cartilage (**Figure 6A**). Notably, the ZnO+US group outperformed single ZnO NPs or US-treated group to restore cartilage layer thickness, reconstruct tidal line structures and delaminate the cartilage-bone interface. The quantitative OARSI score further confirmed that the score of the combined treatment group was 2.9-fold lower than that of the OA group, suggesting that it was effective in stalling the process of OA-arised cartilage degeneration (**Figure 6B**).

To further understand its mechanism, OA-associated markers, i.e., IL-6 and MMP13 in the articular cartilage were investigated through immunohistochemical analysis (**Figure 6C**). In the OA group, the two pro-inflammatory factors were strongly positively expressed around the chondrocytes (with a dense distribution of brown particles) and were significantly regulated by accumulation over time. In contrast, the intensity of positive signals in the ZnO+US group decreased to near-normal levels, suggesting the considerably-inhibited inflammatory cascade response. Semi-quantitative analysis also obtained the identical results, where IL-6 and MMP13 expressions in the combined treatment group received the largest reductions (68% and 47%, respectively) compared with the OA group (**Figure 6D**). Together, these results suggest that US-driven piezoelectric ZnO NPs can achieve cartilage protection and synergistically delay OA progression through the dual action mechanisms, i.e., repairing the structural integrity of the cartilage matrix and targetedly inhibiting IL-6/MMP13 inflammatory axis.

### US-driven sono-piezoelectric ZnO NPs regulated neuroinflammatory reflex-arc mediated α7nAChR-P2RX7 axis

Increased macrophage recruitment and CX3CL1/FKN release were associated with the onset and persistence of OA-correlated pain 51, which was closely related to changes in α7nAChR expression 52. Additionally, α7nAChR has been identified as a key receptor of cholinergic anti-inflammatory pathway and regulates P2RX7 for anti-inflammation 53. Regarding the critical regulatory role of neuroinflammatory reflex arc-mediated α7nAChR-P2RX7 axis in the pathological process of OA, our US-driven piezoelectric ZnO NPs are expected to regulate α7nAChR expression in DRG and suppress downstream inflammatory and pain pathways, because electrical stimulation can upregulate α7nAChR expression and activate the cholinergic anti-inflammatory pathway to inhibit inflammation.

**Figure 7:**
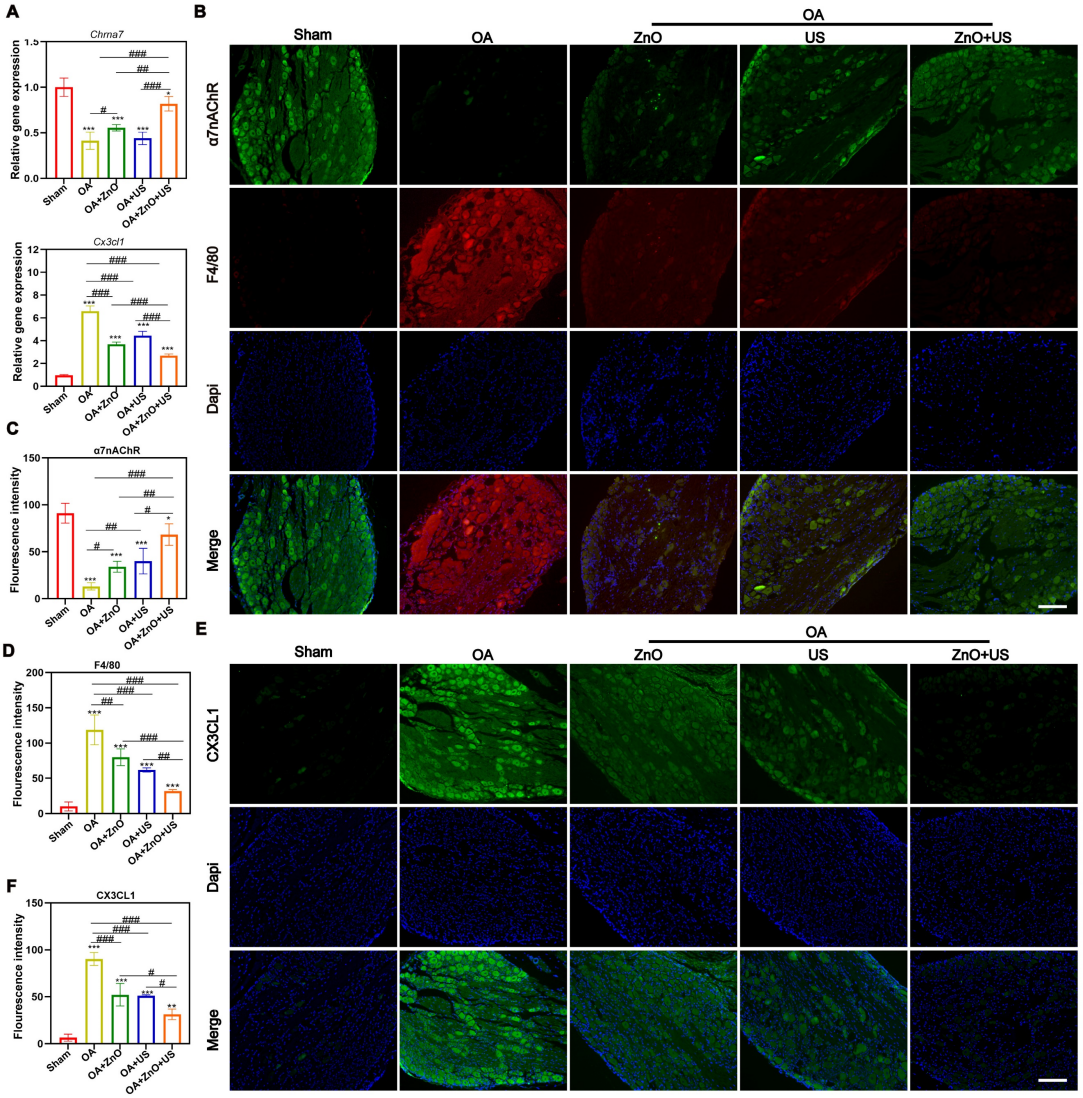
** US-driven piezoelectric ZnO NPs affect the expression of analgesic and anti-inflammatory markers in DRG of OA rats.** (A) mRNA expression levels of *Chrna7* and *Cx3cl1* in DRG. (B) Immunofluorescence staining images for colocalization of α7nAChR and F4/80 in DRG (Scale bar: 200 μm). (C and D) Semi-quantitative analysis of α7nAChR and F4/80 in (B). (E) Immunofluorescence staining images for CX3CL1 in DRG (Scale bar: 200 μm). (F) Semi-quantitative analysis of CX3CL1 in (E). Data is mean ± SD, (n = 5). *p < 0.05, **p < 0.01, ***p < 0.001 *vs.* control; ^#^*p <* 0.05, ^##^*p <* 0.01, ^###^*p <* 0.001 for intergroup comparisons.

To verify them, qRT-PCR tests were carried out. US-driven piezoelectric ZnO NPs in the ZnO+US group was found to significantly up-regulate the expression of *Chrna7* mRNA in the DRG, while significantly suppressing the transcript level of pain-associated chemokine, (e.g., *Cx3cl1*) (**Figure 7A**). The alterations in the gene expression profile of *Chrna7* and *Cx3cl1* suggests that piezoelectric stimulation may remodel the neuroimmune dialogue by activating cholinergic anti-inflammatory pathways. Immunofluorescence co-localization revealed that F4/80 representing macrophages are highly expressed in DRG of OA group, while α7nAChR signal was weak, indicating that macrophage infiltration coincided with neuroreceptor function inhibition under inflammatory environment (**Figure 7B-D**). But in the ZnO+US group, not only significantly enhanced the fluorescence intensity of α7nAChR was significantly elevated, with which the infiltration of F4/80-positive macrophages was effectively hampered, confirming that piezoelectric stimulation synergistically regulated neuronal receptor expression and inflammatory cell recruitment. In addition, immunofluorescence analysis of CX3CL1 protein further corroborated the above findings, and DRG neurons in the OA group showed strong CX3CL1 signals with the surrounding tissues. Nevertheless, CX3CL1 signals were significantly attenuated in the ZnO+US group, which verified the inhibitory effect of sono-piezoelectric current on pain-related neuro-factors at the level of spatial distribution (**Figure 7E,F**). The CX3CL1/FKN-mediated pain signaling inhibition and macrophage-driven neuroinflammatory cascade blockading through the specific activation of α7nAChR receptor in the DRG were encouraged to coordinate dual anti-inflammatory and analgesic efficacy at the level of the neuroimmune crossover. This proof of concept for targeting the neural reflex arc finds a new route to suppress pain and treat OA.

**Figure 8:**
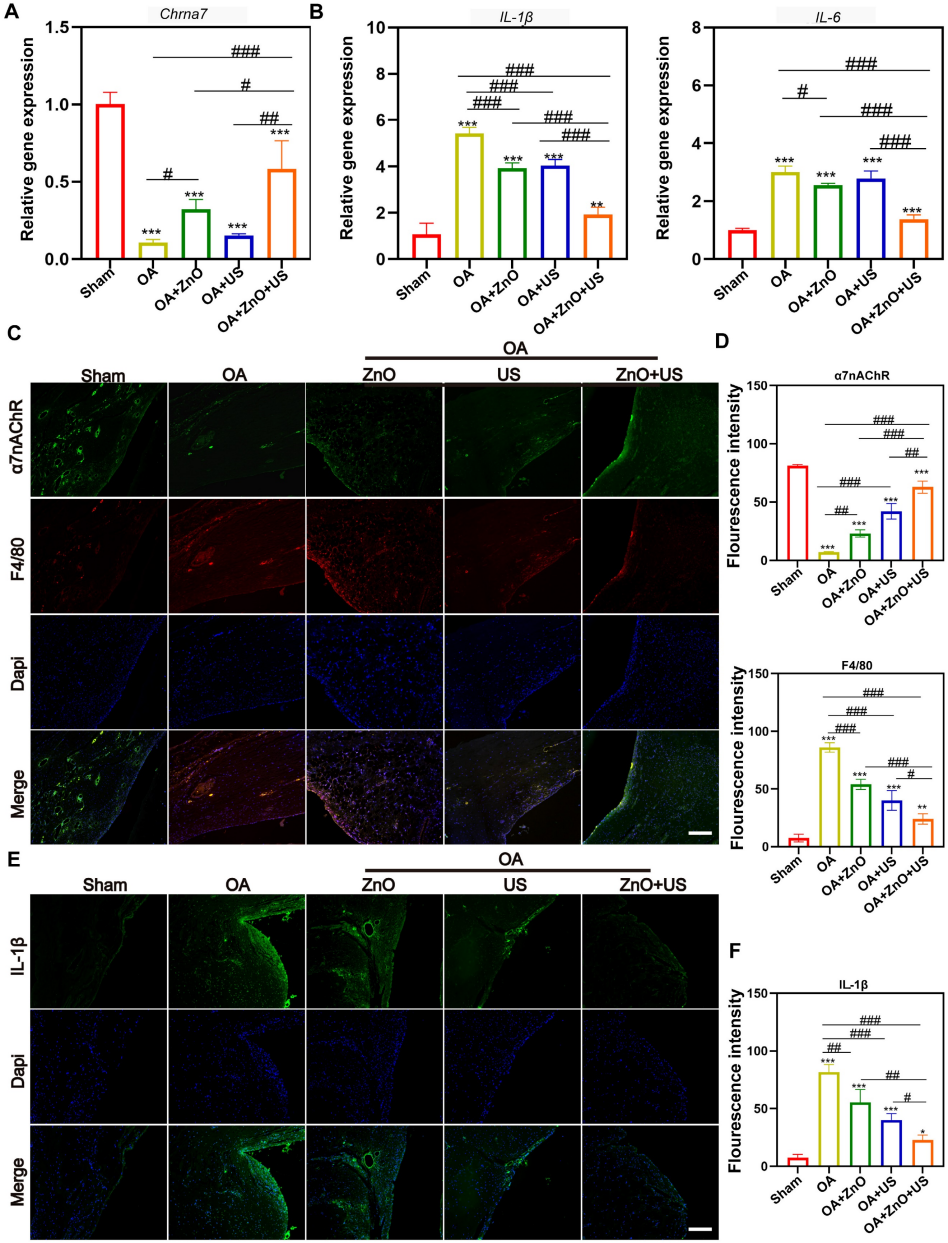
** US-driven piezoelectric ZnO NPs alleviate synovial inflammation in OA rats.** (A,B) mRNA expression levels of *Chrna7* (A), and *IL-1β* and *IL-6* (B) in synovium. (C) Immunofluorescence staining for colocalization of α7nAChR and F4/80 in synovium (Scale bar: 200 μm). (D) Semi-quantitative analysis of α7nAChR and F4/80 in (C). (E) Immunofluorescence staining for IL-1β in synovium (Scale bar: 200 μm). (F) Semi-quantitative analysis of IL-1β in (E). Data is mean ± SD, (n = 5). *p < 0.05, **p < 0.01, ***p < 0.001 *vs.* control; ^#^*p <* 0.05, ^##^*p <* 0.01, ^###^*p <* 0.001 for intergroup comparisons.

Furthermore, we examined the expression of genes and proteins associated with synovial inflammation to deeply understand the molecular mechanisms on how piezoelectric ZnO NPs are driven by US to modulate the neuroinflammatory reflex arc. We focused on the role of α7nAChR-mediated cholinergic anti-inflammatory pathway in synovial tissue. At the gene level, qRT-PCR analysis revealed a significant 4.5-fold elevation in mRNA expression of *Chrna7* in the synovium of the ZnO+US combination treatment group compared to the untreated OA group (**Figure 8A**), while the transcript levels of the pro-inflammatory factors *IL-1β* and *IL-6* were decreased by 64% and 54% respectively (**Figure 8B**). At the cell level *in vitro*, identical results were obtained (**Figure S5**), where the protein levels of IL-1β and IL-6 in the ZnO+US group were the lowest, indicating the translation inhibition by our sono-piezoelectric effects. The gene- and protein-level expression reversal implies that piezoelectric stimulation may directly inhibit downstream inflammatory signaling cascades by activating cholinergic receptors. To verify this hypothesis, we further resolved the cellular phenotype of synovial tissue (**Figure 8C,D**). Results showed that a large number of F4/80^+^ macrophages were infiltrated in the synovial membrane in the OA group and negatively correlated with α7nAChR signals, whereas in the ZnO+US group, the intensity of α7nAChR expression in synovial cell membranes was significantly enhanced, while the density of F4/80⁺ cells was reduced to 28% of that in the OA group. This change in receptor-effector cell dynamics suggests that sono-piezoelectrical signals induced by ZnO+US may directly inhibit macrophage recruitment to inflammatory joints by upregulating α7nAChR. Critically, this immunomodulatory effect was also reflected by pro-inflammatory factor secretion inhibition (**Figure 8E,F**). The synovial cytoplasm in the OA group showed strongly diffused fluorescent signals of IL-1β, while the signal and intensity in the ZnO+US group was attenuated to the control level. This result forms a closed loop with the genetic data, confirming that piezoelectric stimulation doubly inhibited IL-1β transcription and translation via the α7nAChR-P2RX7 axis and ultimately blockaded the positive feedback loop of inflammation. Above multichannel experiments suggest that US-driven piezoelectric ZnO NPs targeted and activated the α7nAChR receptor or signaling axis (a neuroimmune regulatory nexus in synovial tissues) and remodeled joint immune homeostasis by inhibiting synovial macrophage infiltration and attenuating critical pro-inflammatory IL-1β/IL-6 storm. This innovative therapeutic strategy based on “electrical signal-neural reflex-immunomodulation” provides an experimental basis for intervening the neuroinflammatory axis in OA.

**Figure 9:**
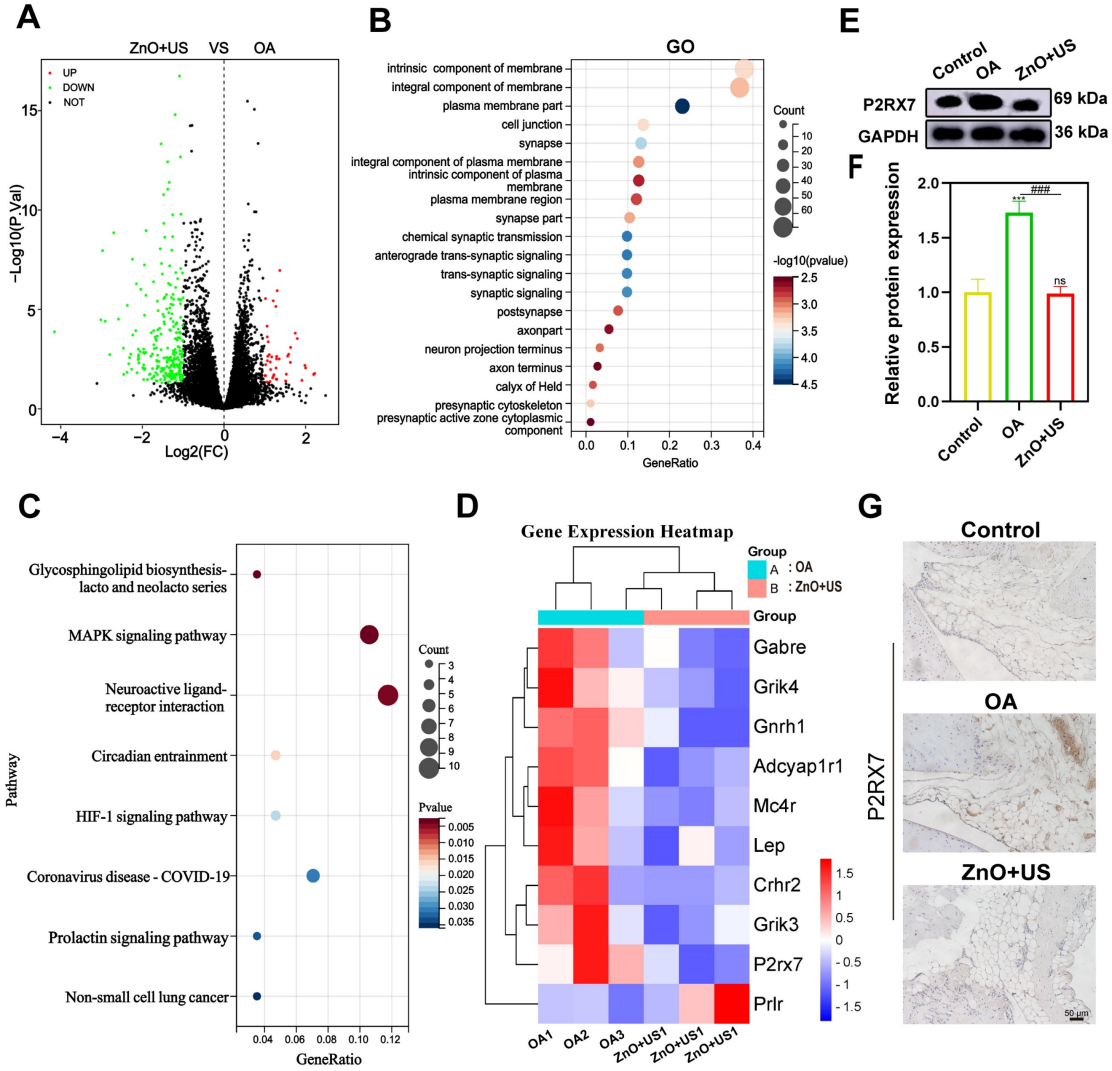
** Transcriptomic analysis of synovium in the treatment of US-driven piezoelectric ZnO NPs.** (A) Volcano plot showing the identified up/downregulated differentially expressed genes (DEGs) after ZnO NPs mediated treatment group versus Control group. (B,C) GO (b) and KEGG (c) analysis of differential gene expression. (D) Heatmap of the top 20 DEGs enriched in neuroactive ligand-receptor interaction pathways. (E) P2RX7 protein expression in synovium that was tested by Western blot. (F) Semi-quantitative analysis of P2RX7 in (E) normalized to GAPDH. (G) Immunohistochemical staining for P2RX7 in synovium (Scale bar: 50 μm). Data is presented as mean ± SD, (n = 3); ^**^p < 0.01 vs. Control.

To thoroughly resolve the anti-inflammatory and analgesic mechanisms of US-driven piezoelectric ZnO NPs, we used transcriptome sequencing technology to systematically compare the gene expression profiles of synovial tissues in both control and ZnO+US groups. Therein, the differential gene expressions in the neuroactive ligand-receptor interaction pathway were highlighted with a specific emphasis on the pivotal regulator P2RX7 (purinergic receptor P2X7) (**Figure 9**). The volcano plot screened out a total of 294 differentially expressed genes based on significant difference criteria (**Figure 9A**). Among them, 42 genes were significantly up-regulated in expression, while 252 genes showed a significant down-regulation trend. This significant changes at the transcriptome level fully demonstrated that ZnO NPs have a broad and far-reaching regulatory effect on inflammation-related pathways. Further analysis of KEGG pathway enrichment revealed that these DEGs were highly enriched in the “neuroactive ligand-receptor interaction” signaling pathway, suggesting that the neuro-immunomodulatory network was affected by the biological effects originating from US-activated sono-piezoelectric ZnO NPs (**Figure 9B,C**).

Notably, P2RX7 had the most significant expression change among the core differential genes, and its down-regulation trend was highly consistent with the pain relief and inflammation suppression phenotypes in the pre-phenotypic results (**Figure 9D**). Regarding this, the suppression of P2RX7 as a pivotal molecule for nociceptive sensitization and activation of inflammatory vesicles might be directly associated with the neuro-modulatory mechanism of US-activated ZnO NPs-mediated sono-piezoelectric effects. To validate this discovery, we inspected P2RX7 expressions at the protein level by Western blot (**Figure 9E,F**) and immunohistochemistry (**Figure 9G**). The expression of P2RX7 in synovial tissues was significantly decreased in the ZnO+US group compared with the OA group, and its spatial distribution was negatively correlated with inflammatory areas. These multi-omics data collectively deciphered that US-driven piezoelectric ZnO NPs may block nociceptive signaling and inflammatory cascade responses by inhibiting the P2RX7-mediated neuroimmune axis, thereby coordinating the dual therapeutic effects of OA at the molecular level. Additionally, *in vivo* metabolic and pathological survey verify the excellent biosafety of ZnO NPs since they failed to cause damages to rat organs and no significant difference of Zn^2+^ in metabolic organs (liver and kidney) (**Figure S6 and 7**), laying a solid foundation to *in vivo* application.

## Conclusion

In summary, US-driven sono-piezoelectric-neuromodulation strategy based on piezoelectric ZnO NPs with dynamic piezoelectric fields (peak value 0.7 V/36 μA) was established to adapt to the deep joints and attenuate osteoarthritic neurogenic inflammation, pain and peripheral tissue damages via programmable bioelectric microenvironments. Therein, the non-invasive US spatial-temporal programming address the bottleneck mattering depth and targeted delivery that current implantable electrical stimulation encountered. The core mechanism lies in the activation of the “sensory neuron-cholinergic anti-inflammatory pathway” reflex arc by piezoelectric field through targeting the α7nAChR-P2RX7 neuroimmune axis in the DRG and synovial tissues. This “physical energy-bioelectricity-neuroimmune” cascade modulation strategy a pioneered paradigm of non-invasively electrical microenvironment programming within deep joint tissues, enabling the precision therapy of OA and other neuroinflammatory diseases. And provide a guidance in building intelligent, piezoelectric immunomodulatory platforms responsive to the pathological microenvironment.

### Experimental section

All details including materials, characterization and experimental methods are included in Supporting information.

## Supplementary Material

Supplementary experimental section, figures, and tables.
