# Supplementary material for: Sono-piezoelectric cues regulate neuroinflammatory reflex-arc-mediated α7nAChR-P2RX7 axis to dampen osteoarthritis-correlated pain with osteoarthritis attenuation
Source: Theranostics. 2026 Jan 30;16(8):4358–73. doi: 10.7150/thno.125686 (PMC12905820; doi:10.7150/thno.125686)
Supplement: Supplementary file 1 — Supplementary experimental section, figures, and tables. [file thnov16p4358s1.pdf]

Supporting Information for

Sono-Piezoelectric Cues Regulate Neuroinflammatory Reflex-Arc-Mediated α7nAChR-P2RX7 Axis to Dampen Osteoarthritis-Correlated Pain With Osteoarthritis Attenuation

Qiuling Zhong,[1,#] Junqi Xie,[1,2,#] Maocheng Zuo,[3,4,#] Chuanan Liao,[5,#] Qiuxia Peng,[4,5] Simeng Yu,[1] Pan Hu,[1] Yuanyuan Liu,[1,2] Li Zheng,[1,2,*] Kun Zhang,[4,5,*] Zhenhui Lu,[1,2,*] and Jinmin Zhao[1,6]

Table of Contents

Supplementary Experimental Section……………………………………………………………………………..S3

Supplementary Tables Section...………………………………………. ……………………………………….S9

Supplementary Figures Section……………………………………... ………………………………………...S10

Supplementary References ……………………………………………. ………………………………………S15

Experimental section

Materials

Zinc oxide ($Zn(NO_3)_2 \cdot 6H_2O$, ≥99.8%) and sodium hydroxide (NaOH, ≥99.8%) were purchased from Aladdin Chemical Reagent Co., Ltd (Shanghai, China). *N*,*N*-dimethylformamide (DMF) (≥99.5%) and dimethyl sulfoxide (DMSO) were purchased from Macklin Biochemical Technology Co., Ltd (Shanghai, China). All chemical regents were used directly without any further purification.

Preparation of ZnO NPs

ZnO NPs were synthesized using microfluidic technique. Briefly, the extension tube of the micropump was made into a Y-shaped tube, divided into two ports, A and B, which were connected to two syringe ports respectively: port A was connected to a syringe containing 300 mM $Zn(NO_3)_2$ solution, and port B was connected to a syringe containing 125 mM NaOH solution. The solution in port A is injected at 50 μL/min and the solution in port B is injected at 100 μL/min. Both were pumped into the Y-type micro-pump channel. Both were pumped through the pump into a Y-shaped microchannel. The synthesis product was collected at the outlet in a 50 mL round-bottomed flask and then placed directly into a water bath at 80 °C for 1 h of continuous heating to complete the crystallization. After cooling, it was centrifuged and then purified by centrifugation. The supernatant was removed. After washing three times with ultrapure water, the resulting white product was placed in a vacuum drying oven for 12 h to obtain high-purity ZnO NPs.

Characterization of ZnO NPs

The morphology, distribution and size of ZnO NPs were analyzed by TEM (H-7650, Hitachi, Japan) and DLS (Malvern Panalytical, UK). The element composts were analyzed by high-resolution Mappingas well as X-ray energy spectroscopy EDX (Thermo Scientific™ Talos F200S 200kV S/TEM, USA). Infrared spectrum and X-ray diffraction characteristics were measured by FTIR (Nicolet IS50R, USA) and XRD (Rigaku, Japan).

Piezoelectric performance

ZnO NPs was dissolved in ultrapure water and sonicated for 30 min at 20 °C. Appropriate amount of the solution was added drop wise to bottom electrode that coated with 100 nm thickness of Pd. The sample was dried and polarized at room temperature for 30 min with a voltage of 8 kV. Then the sample was coated with a 5 nm thickness of Pd as the upper electrode. The piezoelectric properties of ZnO NPs were examined by a PFM (Oxford, Shanghai, China).

The piezoelectric properties of ZnO NPs were further analyzed by a constructing a piezoelectric film based on PVDF. Firstly, ZnO particles (30 wt %, relative to PVDF) were added to DMF solution, and the mixture was sonicated for 30 min to ensure a homogeneous mixture. PVDF powder was finally blended into the prepared solvent and the solution stayed in 60 ˚C hot water bath for 6 h with stirring. Electrospinning was carried out at a push rate of 0.1 mm/min at an electrospinning distance of 15 cm between the spinneret and the collector. The electrospinning voltage was 20 kV. Pristine PVDF nanofiber membranes were prepared under the same conditions. All the nanofiber membranes were stored in a dry cabinet. A conductive copper foil was attached to the surface of the nanofiber membrane and two wires were connected externally for stimulation using ultrasound. The output voltage and current of ZnO NPs were recorded with an oscilloscope (TBS 1102, Tektronix, USA).

Cell isolation and culture

Chondrocytes were extracted from knee cartilage of Sprague-Dawley (SD) rats (3~5 days old) by collagenase type II. Cells were cultured with DMEM medium (Gibco, USA) containing 10% fetal bovine serum (FBS; Every Green, Hangzhou, China), and 1 % penicillin–streptomycin solution (Biosharp, Shanghai, China) in an incubator (37 ˚C, 5 % CO_2, Thermo Fisher Scientific, USA). Chondrocytes at passage two were used and the morphology was observed by a optical microscope. Synoviocytes were purchased from Wuhan Procell Life Sciences Co (CM-R083).

Cell counting kit-8 (CCK-8) assay

Cells seeded on 96-well-plates were stimulated with or without 10 ng/mL of IL-1β. After treated with different concentrations of ZnO or ultrasonic intensity/time for 72 h, cultured medium was removed and added with fresh medium. Then 10 μL of CCK-8 (Biosharp, China) was added to each well and incubated at 37 ˚C for 4 h. The absorbance at 450 nm was measured with a microplate reader (Thermo Fisher Scientific, USA).

Cell treatment

Cells were divided into five groups: (1) Control: cells cultured with complete medium; (2) IL-1β: cells stimulated with IL-1β (10 ng/mL); (3) ZnO: IL-1β (10 ng/mL) induced cells cultured with 10 μg/mL of ZnO; (4) US: IL-1β (10 ng/mL) induced cells combined ultrasound therapy (0.35 W/cm^2, 90 s); (5) ZnO+US: IL-1β (10 ng/mL) induced cells cultured with 10 μg/mL of ZnO combined ultrasound therapy (0.35 W/cm^2, 90 s). All the IL-1β induced cells were pre-treated with IL-1β for 1

h followed by the experimental treatments. And the cells were harvested 24 h post-treatment.

Live/dead cell staining

After washing with PBS for three times, Live and dead cells were stained with a Calcein-AM/propidium iodide (PI) kit (Beyotime, China) for 5 min in the dark. Rinse by PBS again and the images were captured by a fluorescence microscope (Olympus, Japan). Semi-quantitative analysis for the images was calculated by Image J software.

Deoxyribonucleic acid (DNA) and glycosaminoglycan (GAG) quantitation

Digested cells were collected and added with Proteinase K (Beyotime, Shanghai, China) and incubated in the 56°C for 16 h. DNA was measured by dying with Hoechst 33258 (Solarbio, Beijing, China) and detected by a fluorescence microplate reader (BioTek Synergy H1). Calf thymus DNA (Solarbio, Beijing, China) was used as a standard. GAG was measured by staining with DMMB (Sigma-Aldrich, USA) with chondroitin sulfate (Aladdin, Shanghai, China) as standard. Relative amount GAG was normalized to the total DNA.

Scratch wound healing assay

A scratch-wound assay was used to detect the cell migration. Cells cultured in the 12-well-plates until reaching too fully confluent. A scratch was made by a sterile pipette tip on the chondrocytes. After different treatment for 24 h, the images of the scratches were obtained by a microscopy.

Toluidine blue staining

The ECM production of chondrocytes was measured by toluidine blue staining. After removing the culture medium, cells were washed with PBS and incubated with toluidine blue dye (Aladdin, China) in room temperature for 10 min. The excess dye was then washed by PBS and the images were captured using a microscopy.

Quantitative real-time polymerase chain reaction (qRT-PCR) analysis

Total RNA was purified using a total RNA kit (Magen, Guangzhou, China). The RNA was then reverse transcribed into cDNA using a first-strand cDNA synthesis kit (Takara, Beijing, China). Primer sequences used in this study are listed in Table S1. A SYBR Green qRT-PCR Super Mix Plus system (Roche, Switzerland) was used for amplification detection under follow conditions: the reaction consists of three major steps: denaturation, annealing, and extension, and the reaction conditions are preheating at 95 °C, reacting at 95 °C for 10 s and 60 s (40 cycles) at 95 °C and 60 °C, respectively, and reacting at 65 °C for 10 s. The relative expression of the genes was calculated by

the $2^{-\Delta\Delta CT}$ method and glyceraldehyde-3-phosphate dehydrogenase (GAPDH) was used as an internal control.

Establishment of rat OA model

All animal experiments were conducted in accordance with the guidelines of the Animal Research Ethics Committee of Guangxi Medical University. Fifty-four SD rats (male, 6-8 weeks age) were used in this study for in vivo study. Rats were intraperitoneally injected with pentobarbital sodium (40 mg/kg) for anesthesia. Then an anterior cruciate ligament tenotomy (ACLT) surgery was conducted to establish an OA model. The sham-operated group used the same surgical method but did not cut the ACL. Four weeks later, rats were divided randomly into five groups: (1) sham operation group; (2) OA group: OA rats intra-articular injection of 0.5 mL PBS; (3) ZnO group: OA rats intra-articular injection of 0.5 mL ZnO NPs; (4) US group: OA rats intra-articular injection of 0.5 mL PBS combined ultrasound therapy (0.35 W/cm^2, 90 s) therapy; (5) ZnO+US group: OA rats intra-articular injection of 0.5 mL ZnO NPs combined ultrasound therapy (0.35 W/cm^2, 90 s) therapy. The treatment of US was performed every two days.

Pain behavior and gait patterns

The mechanical sensitivity to pain was assessed using the Von Frey nociceptive test. The paws of rats were stimulated using Von Frey hairs test (Yuyuan Scientific Instrument Co., Shanghai, China) with different diameters and stiffness. Sufficient time intervals were left between the different filaments stimulated to allow the experimental animal to return to the basal state. Observe the responses of the experimental animals to the different stimuli, such as lifting their feet or making noises, to determine their pain sensitivity. We repeated the test three times and recorded the results of each test.

The gait of rats was recorded under non-stimulated conditions using an animal visual gait analysis system (VisuGait XR-FP101, Xinruan Information Technology Co., Shanghai, China) to assess motor deficits and pain-induced gait changes. Rats can walk from one end of the walking platform to the other. The system uses a unique footprint light refraction technology to capture real footprint footprints through a high-speed HD camera placed underneath the walking platform, which are then automatically categorized by the computer vision processing software. At the same time, the system is able to detect differences in the relative pressure of the footsteps, which is the result of the different distribution of the animal's weight across its four paws as it walks.

Macroscopic and histological evaluation

After 4 and 8 weeks of treatment, the experimental animals were euthanized. Knee joint specimens from all groups were collected and photographed for macroscopic observation. The macroscopic score was performed through 3 valuators who were blinded to the groups according to the scales of 0–4 [1]. Knee joints, DRG and main organs (hearts, livers, spleens, lungs and kidneys) were collected and fixed with 4% paraformaldehyde (biosharp, China) for 48 h. Then joints were decalcified in 10% ethylenediaminetetraacetic acid decalcification (ETDA; Aladdin, Shanghai, China) for 30 d. Samples were embedded in paraffin and cut into sections (4 μm thickness). The joints sections were dewaxed and stained with H&E (Solarbio, Beijing China) or safranin O/fast green (Solarbio, Beijing China). Images were taken by a light microscope (BX 63, Olympus, Japan). The histological score was performed blindly by three observers according to the method [2]. The sections of main organs were stained with H&E to evaluate the toxicity of NPs in vivo.

Immunohistochemical and immunofluorescence staining

Fixed cells and dewaxed sections of joints and DRG were rinsed with PBS and incubated with 3% H_2O_2 (ZSGB-BIO, Beijing, China) at 37 °C for 15 min. The samples were then blocked with goat serum (ZSGB-BIO, Beijing, China) for 20 min and followed by incubating with the primary antibodies: Col2A1 (28459-1-AP, 1:100, Proteintech, Wuhan, China), IL6 (21865-1-AP, 1:100, Proteintech, China), TNF-α (17590-1-AP, 1:100, Proteintech, Wuhan, China), MMP13 (18165-1-AP, 1:100, Proteintech, Wuhan, China), CHRNA7 (TA374764S, 1:100, Origene, Wuxi, China), F4/80 (28463-1-AP, 1:100; proteintech, Wuhan, China), CX3CL1 (#AF0129, 1:100, affinity, USA) and P2RX7 (11144-1-AP, 1:100; proteintech, Wuhan, China) antibodies overnight at 4°C. For the immunohistochemical staining, sections were successively incubated with secondary antibody (ZSGB-BIO, Beijing, China) and developed by diaminobenzidine (DAB) kit (ZSGB-BIO, Beijing, China). After stained by hematoxylin, the images were obtained. For immunofluorescence staining, samples were incubated with fluorescence secondary antibodies at 37 °C in the dark for 1 h. Nuclear were stained with DAPI (Solarbio, Beijing, China) at room temperature protected from light. A fluorescence microscope was used for capturing the photographs.

Transcriptome sequencing

Transcriptome RNA sequencing was performed using the synovium tissues from rats in OA and ZnO+US groups. RNA purification, reverse transcription, library construction and sequencing were

performed at Shanghai Majorbio Bio-pharm Biotechnology Co., Ltd. (Shanghai, China) according to the manufacturer's instructions. After quantified by Qubit 4.0, the sequencing library was performed on DNBSEQ-T7 platform (PE150) using DNBSEQ-T7RS Reagent Kit (FCL PE150). The raw paired end reads were trimmed and quality controlled by fast with default parameters. Then clean reads were separately aligned to reference genome with orientation mode using HISAT2 software. The mapped reads of each sample were assembled by StringTie in a reference-based approach [3]. In R, genes with $P<0.05$ and $|\log2FC|\geq2$ are considered differentially expressed genes (DEGs). Additionally, GO and KEGG pathway enrichment analyses were performed using R to examine pathways associated with the DEGs [4].

Western blotting

The synovium tissues were lysed with RIPA lysis buffer, and the concentrations of total proteins was detected by using a BCA protein kit (Beyotime, Shanghai, China). Then the proteins were separated by 12% SDS-polyacrylamide gel electrophoresis and transferred onto the PVDF membranes. The membranes were incubated with the primary antibodies against P2RX7 (28207-1-AP, Proteintech, Wuhan, China) and GAPDH (10494-1-AP, Proteintech, Wuhan, China). After exposure to secondary antibody (), the membranes were scan by a high-sensitivity imaging instrument (GE, USA) and the densities and gray levels of all bands were quantified with Image J (2.x) software.

Measurement of tissue Zn^{2+} content

Cartilage, liver and kidney was collected after 4 and 8 weeks of treatment, and then 0.1 of tissues were weighted. After homogenat and lysis of tissues, the content of Zn^{2+} were detected by using a Zn^{2+} Content Detection Kit (NJDULY, Nanjing, China).

Statistical analysis

All data were expressed as mean± SD (standard deviation). Statistical analysis was performed using GraphPad prism software. One-way analysis of variance (ANOVA) was performed to analyze multiple comparisons.

Supplementary table

Table S1. Primer sequences used in qRT-PCR analysis

Gene	Forward sequence (5' to 3')	Reverse sequence (5' to 3')
GAPDH	TCCAGTATGACTCTACCCACG	CACGACATACTCAGCACCAG
Col2a1	TGCTGGAAAACCTGGTGATG	GTAACCTCTGTGACCCTTGAC
ACAN	GAATGGGAGCCAGCCTACAC	GAGAGGCAGAGGGACTTTCG
IL-6	GGCATGACTCTCACAATGCG	ACAGTGCATCATCGCTGTTC
MMP13	GGACAAAGACTATCCCCGCC	GGCGGGGATAGTCTTTGTCC
Chrna7	GCAAAGAGCCATACCCAG	GCAGGCAGCAAGAATACC
Cx3cl1	TGGTGGCAAGTTTGAGAA	TGGGAAATAGCAGTCGGT
IL-1β	CTTCAGGCAGGCAGTATCACTC	TGCAGTTGTCTAATGGGAACGT
TNF-α	GAGTGACAAGCCTGTAGCC	CTCCTGGTATGAGATAGCAA

Supplementary figures

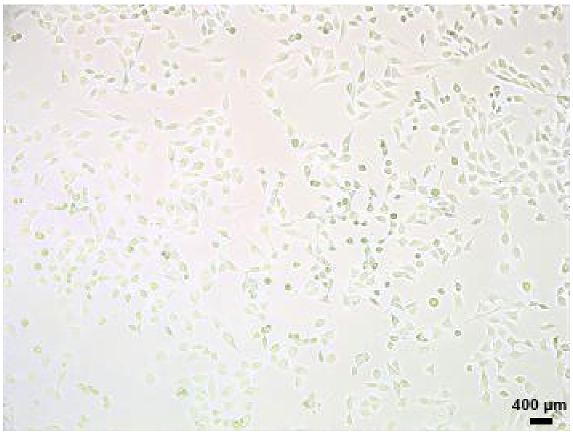

Figure S1. The morphological observations of primary chondrocytes (Scale bar: 400 μm)

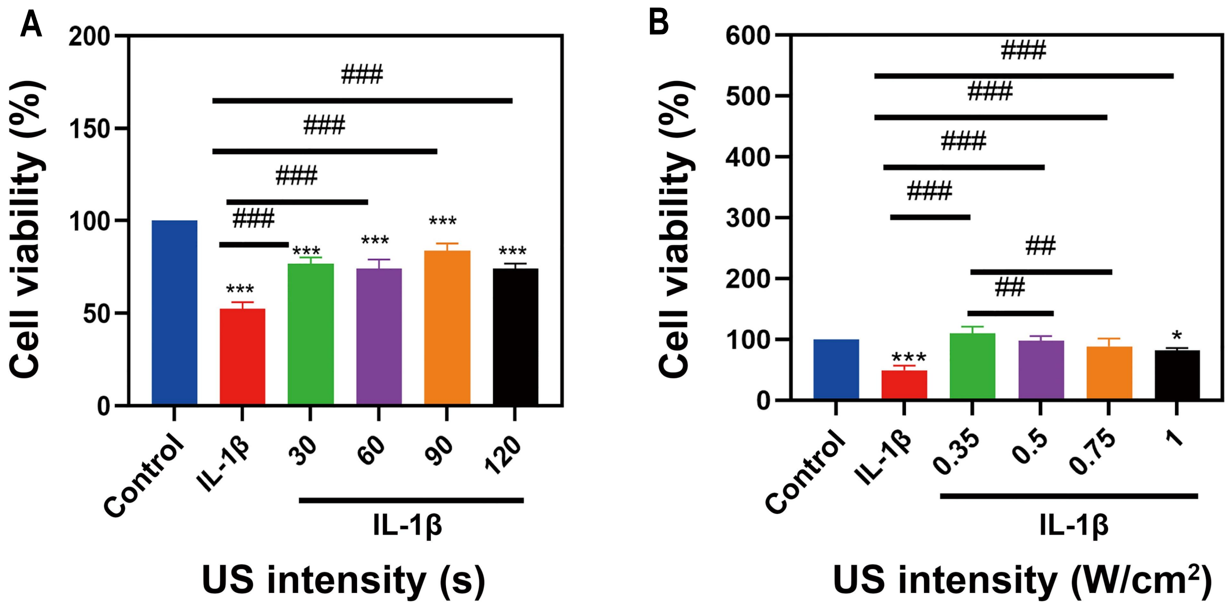

Figure S2. Optimization of ultrasound parameters for piezoelectric ZnO NPs treatment. (**A, B**) Relative viabilities of IL-1β-induced chondrocytes treated with ZnO NPs under varying durations (30–120 s) and ultrasound intensities (0.35–1 W/cm^2). Data represent mean± SD, (n=3). $^{*}p<0.05$, $^{**}p<0.01$, $^{***}p<0.001$ *vs*. control; $^{\#}p<0.05$, $^{\#\#}p<0.01$, $^{\#\#\#}p<0.001$ for intergroup comparisons.

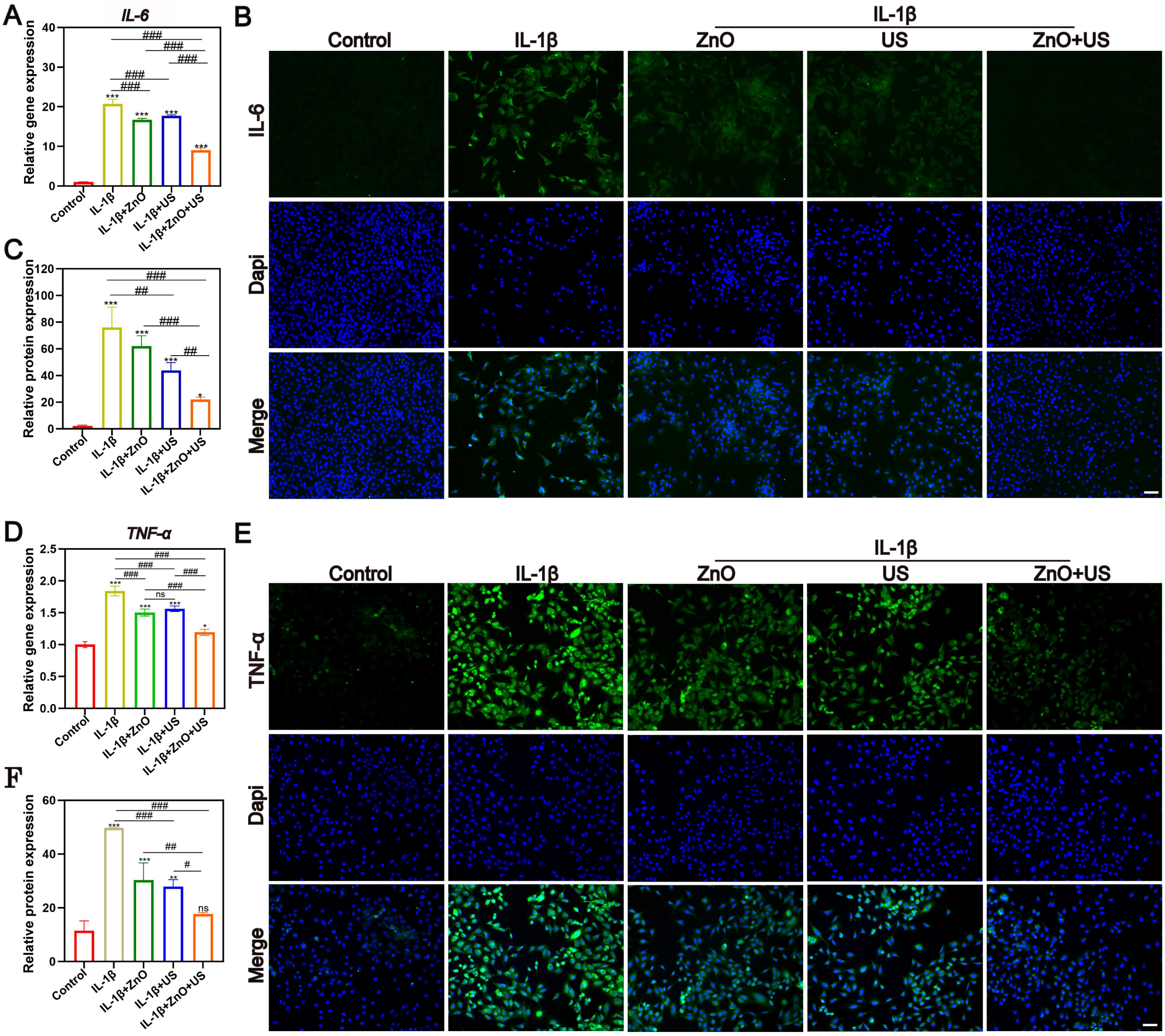

Figure S3. Anti-inflammatory effects of ultrasound-driven piezoelectric ZnO NPs in chondrocytes. (A) qRT-PCR analysis of *IL-6* mRNA expression in chondrocytes across treatment groups. (B) Representative immunofluorescence images of IL-6 (green). (C) Semi-quantitative analysis of immunofluorescence staining for IL-6 (Scale bar: 200 μm) in (B). (D) qRT-PCR analysis of *TNF-α* mRNA expression in chondrocytes across treatment groups. (E) Representative immunofluorescence images of TNF-α (green). (F) Semi-quantitative analysis of immunofluorescence staining for TNF-α (Scale bar: 200 μm) in (E). Data are mean ± SD, (n=3). $^{*}p<0.05$, $^{**}p<0.01$, $^{***}p<0.001$ vs. control; $^{\#}p<0.05$, $^{\#\#\#}p<0.001$ for inter group comparisons.

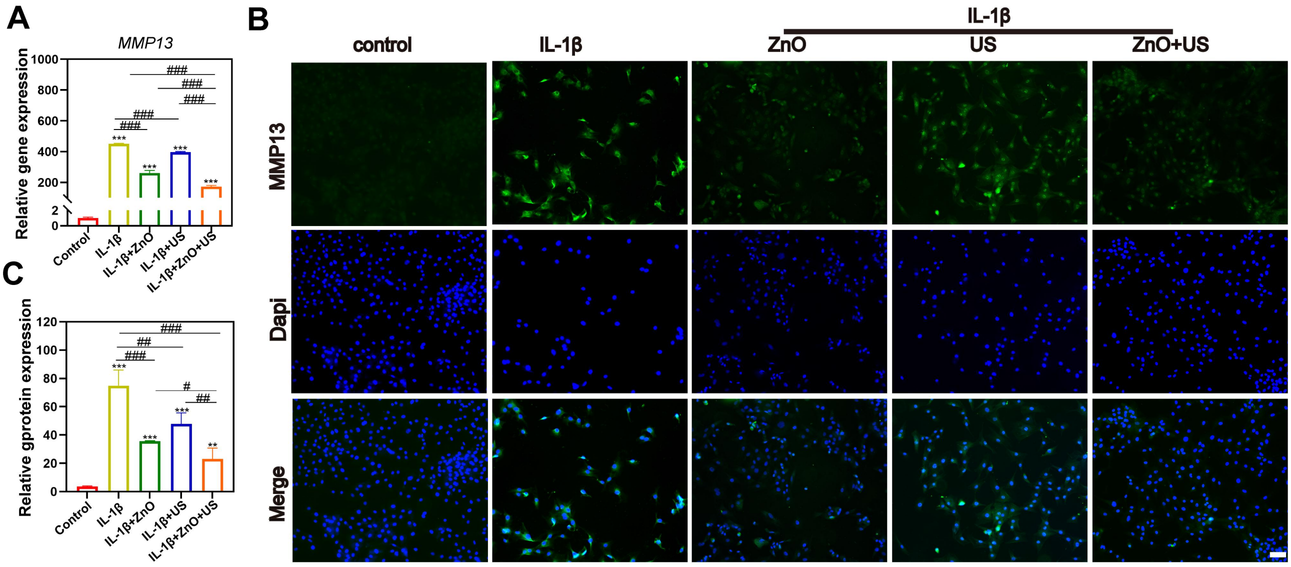

Figure S4. Anti-inflammatory effects of ultrasound-driven piezoelectric ZnO NPs in chondrocytes. (A) qRT-PCR analysis of *MMP13* mRNA expression in chondrocytes across treatment groups. (B) Representative immunofluorescence images of MMP13 (green). (C) Semi-quantitative analysis of immunofluorescence staining for MMP13 (Scale bar: 200 μm) in (B). Data are mean ± SD, (n=3). $^{**}p$<0.01, $^{***}p$<0.001 vs. control; $^{\#}p$<0.05, $^{\#\#}p$<0.01, $^{\#\#\#}p$<0.001 for intergroup comparisons.

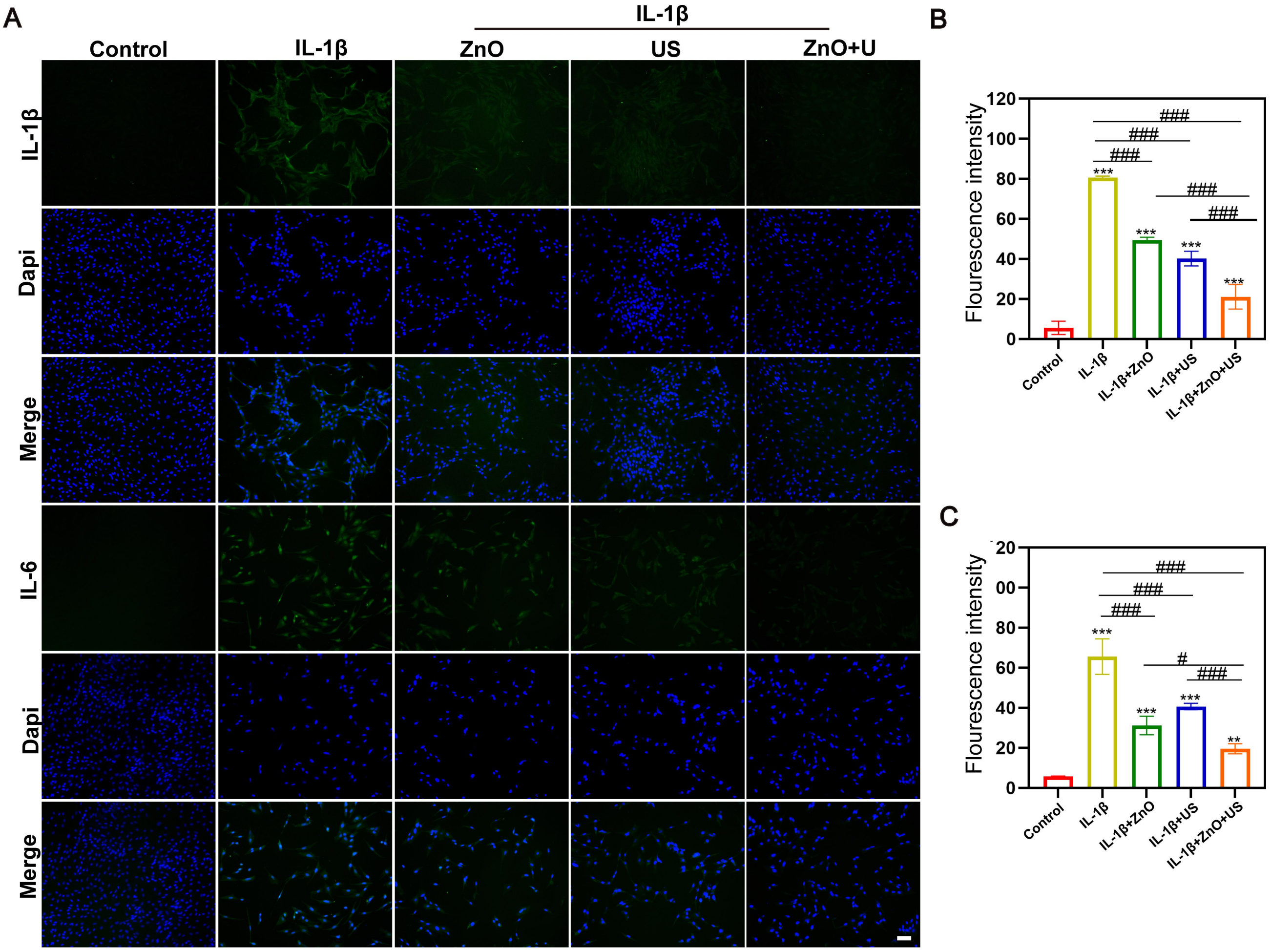

Figure S5. Anti-inflammatory effects of ZnO NPs in synovioblast. (A) Immunofluorescence staining of IL-1β and IL-6 in synovioblast (Scale bar: 200 μm). (B,C) Semi-quantitative analysis of IL-1β (B) and IL-6 (C) fluorescence intensity in (A). Data are mean ± SD, (n=3). $^{**}p<0.01$, $^{***}p<0.001$ vs. control; $^{\#}p<0.05$, $^{\#\#\#}p<0.001$ for intergroup comparisons.

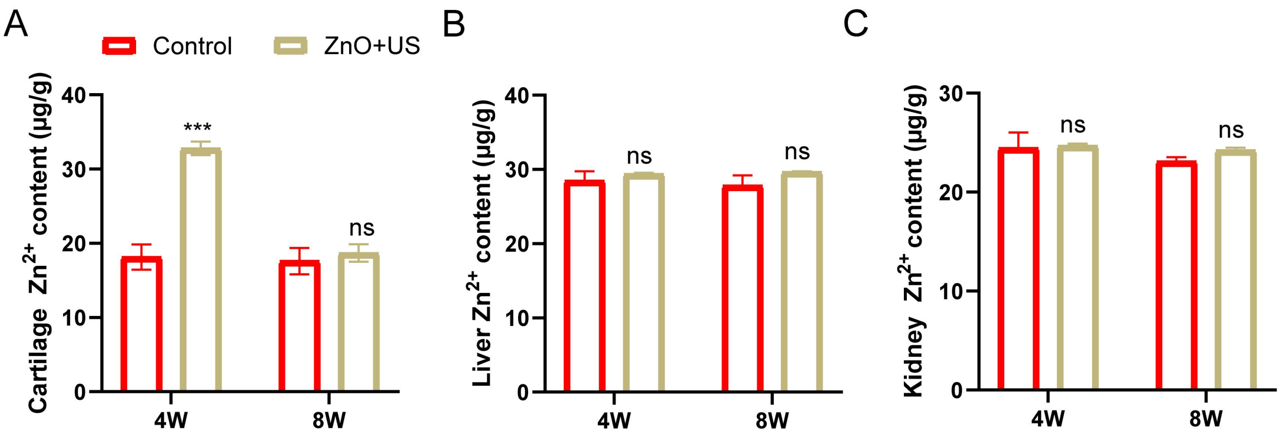

Figure S6. The concentration of Zn^{2+} in cartilage (A), liver (B) and kidney (C) tissue after 4 and 8 weeks of treatment. Data are presented as the mean ± SD, (n = 3); $^{***}p < 0.001$.

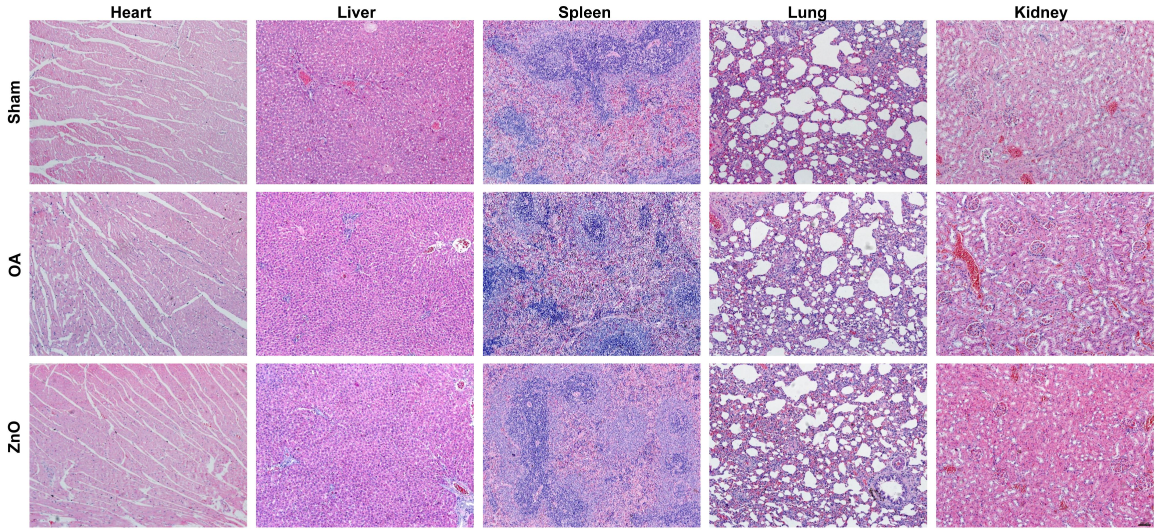

Figure S7. H&E staining of major organs (heart, liver, spleen, lung, kidney) from rats after 8 weeks of treatment (Scale bar: 100 μm). No pathological abnormalities were observed.

References

[1] L. Assi, A. Harrison, A. Kuttapitiya, F. Howe, V. Ejindu, C. Heron, et al. Relation of radiographic severity of knee osteoarthritis to clinical pain scores: Results from the pain perception in osteoarthritis study. Osteoarthritis Cartilage. 2018; 26: S223-4.

[2] K. Nagira, S. Miyagi, Y. Ikuta, M. Lotz. Histological scoring system for periarticular bone changes of the osteoarthritis. Osteoarthritis Cartilage. 2018; 26: S85-6.

[3] E.A. Stahl, D. Wegmann, G. Trynka, J. Gutierrez-Achury, R. Do, B.F. Voight, et al. Bayesian inference analyses of the polygenic architecture of rheumatoid arthritis. Nat Genet. 2012; 44(5): 483-9.

[4] N.C. Butterfield, K.F. Curry, J. Steinberg, H. Dewhurst, D. Komla-Ebri, N.S. Mannan, et al. Accelerating functional gene discovery in osteoarthritis. Nat Commun. 2021; 12(1): 467.
